# Wireless Measurement of Elastic and Plastic Deformation by a Metamaterial-Based Sensor

**DOI:** 10.3390/s141019609

**Published:** 2014-10-20

**Authors:** Burak Ozbey, Hilmi Volkan Demir, Ozgur Kurc, Vakur B. Erturk, Ayhan Altintas

**Affiliations:** 1 Department of Electrical and Electronics Engineering, Department of Physics, UNAM—Institute of Materials Science and Nanotechnology, Bilkent University, Ankara TR-06800, Turkey; E-Mails: volkan@bilkent.edu.tr (H.V.D.); vakur@ee.bilkent.edu.tr (V.B.E.); altintas@ee.bilkent.edu.tr (A.A.); 2 School of Electrical and Electronic Engineering, School of Physical and Mathematical Sciences, Nanyang Technological University, Singapore 639798, Singapore; 3 Department of Civil Engineering, Middle East Technical University, Ankara TR-06800, Turkey; E-Mail: kurc@metu.edu.tr; 4 Faculty of Engineering and Natural Sciences, Abdullah Gul University, Kayseri 38039, Turkey

**Keywords:** displacement sensor, strain sensor, elastic-plastic region, metamaterial, structural health monitoring

## Abstract

We report remote strain and displacement measurement during elastic and plastic deformation using a metamaterial-based wireless and passive sensor. The sensor is made of a comb-like nested split ring resonator (NSRR) probe operating in the near-field of an antenna, which functions as both the transmitter and the receiver. The NSRR probe is fixed on a standard steel reinforcing bar (rebar), and its frequency response is monitored telemetrically by a network analyzer connected to the antenna across the whole stress-strain curve. This wireless measurement includes both the elastic and plastic region deformation together for the first time, where wired technologies, like strain gauges, typically fail to capture. The experiments are further repeated in the presence of a concrete block between the antenna and the probe, and it is shown that the sensing system is capable of functioning through the concrete. The comparison of the wireless sensor measurement with those undertaken using strain gauges and extensometers reveals that the sensor is able to measure both the average strain and the relative displacement on the rebar as a result of the applied force in a considerably accurate way. The performance of the sensor is tested for different types of misalignments that can possibly occur due to the acting force. These results indicate that the metamaterial-based sensor holds great promise for its accurate, robust and wireless measurement of the elastic and plastic deformation of a rebar, providing beneficial information for remote structural health monitoring and post-earthquake damage assessment.

## 1. Introduction

### 1.1. Background

In recent years, wireless and passive sensors have come into prominence owing to their ability to eliminate the need for a power supply, as well as wires or cables for operation. Especially, their use for structural health monitoring (SHM) has been emerging as a promising technique, since they allow for nondestructive testing in structures. The wireless passive sensors employed in SHM are diverse and vary widely in terms of their architecture and operation. They have been recently classified into two groups by Deivasigamani *et al.* [1]; the first group consisting of designs based on oscillating circuits with components, such as capacitors, inductors and resistors [2–4] and the second group including radio frequency identification (RFID) tags, antennas, RF transducers and metamaterial resonators [5–12]. Utilizing metamaterials in sensors can offer higher sensitivity and resolution compared to traditional structures through their stronger localization of fields [13]. They also reduce the sensor size to a great extent compared to a regular structure of which the dimensions are more or less proportional to the operating wavelength. Examples of the employment of metamaterials in sensing include biosensors [14,15], thin-film sensors [16] and wireless strain sensors [6,7,17–21].

Structural health monitoring (SHM) is one of the major areas where the role of the wireless passive sensors is very critical. Steel reinforcing bars (rebars) in reinforced concrete structural members, such as columns and beams, are under tensile or compressive stresses that cause elongation and contraction of each rebar. The changes in the length of rebars of structural members under design loads are usually on the microstrain level, and they occur in the elastic or linear region of the stress-strain curve of a rebar, in which the deformation is not permanent. Monitoring of these slight displacements is vital to understand the level of loading to which a certain structural member is subjected. Readily used instruments to measure strain and displacement, like strain gauges, linear variable differential transformers (LVDT's) and extensometers, are cabled devices, and they are not suitable to be utilized in a real-life scenario inside the concrete. Therefore, it is important that a wireless, passive and accurate measurement scheme is developed. Furthermore, in the case of an overloading or an earthquake, exposure to a high level of force causes damage in the reinforced concrete member, in other words, plastic deformation at the rebars. In plastic deformation, the relation between the stress and strain becomes nonlinear, and the induced damage becomes irreversible. In this region, strain levels as high as 10% can be observed in rebars. Hence, the ability to measure deformation both in the elastic and the plastic region of a steel rebar requires a sensor with a very high sensitivity and extremely wide dynamic range. In other words, a displacement sensor should be able to detect the displacements on the μm level for the elastic range, together with the displacements as large as a few mms in the plastic range.

### 1.2. Metamaterial-Based Displacement Sensor

**Figure 1.**
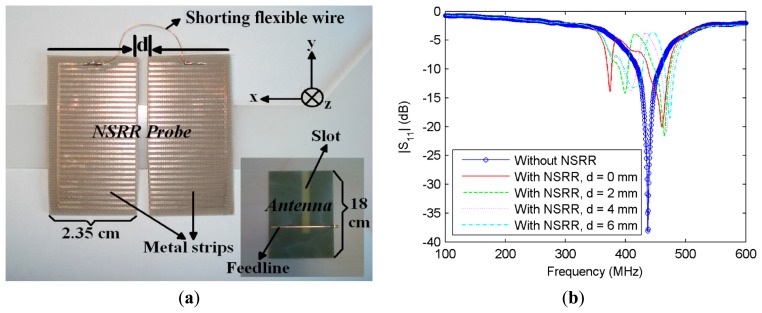
(**a**) Comb-like nested split ring resonator (NSRR) probe geometry separated into two symmetrical parts, which are electrically connected by a long flexible wire. The microstrip single slot antenna is shown at the right bottom (inset); (**b**) Experimental |S_11_| data plotted for the cases when the NSRR is not present in front of the antenna (only the antenna resonance is present) and when the NSRR sensor is present with displacement *d* = 0, 2, 4 and 6 mm, respectively.

**Figure 2.**
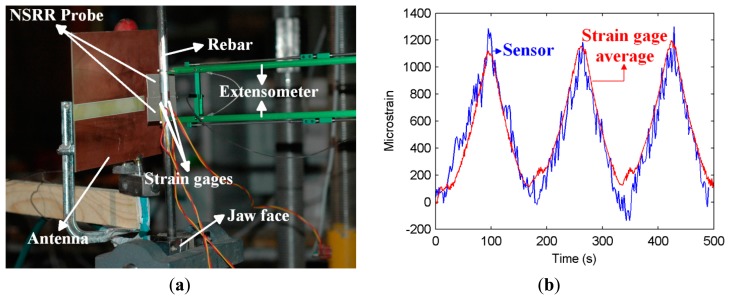
(**a**) High-force mechanical setup on which an 8-mm diameter rebar is pulled and released with a linearly varying force; (**b**) strain measured in time by the metamaterial-based sensor and the strain gauges (showing here the average strain gauge data).

Recently, a displacement sensor, which comprises a metamaterial-based probe and an antenna transmitting/receiving power to/from the probe has been devised by our group [22]. The metamaterial-based probe is a modified version of the comb-like nested split ring resonator (NSRR) geometry, first proposed in [23]. This geometry is made of several pairs of metal strips in parallel (teeth) used to tune the operation resonance frequency by altering the effective capacitance of the sensor. The comb-like NSRR geometry used in this work is shown in Figure 1a. Details related to the design and physical dimensions are explained in [22]. The NSRR probe is located in the near-field of the microstrip single-slot antenna (shown in the Figure 1a inset and Figure 2a), inspired by the design given in [24]. Together, the NSRR probe and the antenna constitute a coupled system, whose resonance frequency changes with the displacement (*d*) on the NSRR probe. The frequency peaks shifting with *d* of the two parts of the NSRR are shown in Figure 1b. The strong coupling is due to the excitation method of the NSRR probe. Since the slot antenna is excited through a microstrip line along the *x*-direction at the back side of the substrate (see Figure 1a), it transmits an x-polarized E-field from the slot introduced at the other side of the substrate. When this field illuminates the NSRR, a strong coupling forms between the antenna and the probe, because the splits between the metal strips of the NSRR probe are also along the *x*-direction. When the antenna polarization is changed, e.g., when the antenna is rotated 90° around the *z*-axis, the resulting polarization yields lower depth resonances and a changed resonance frequency, due to much poorer coupling. Therefore, the antenna orientation must be set accordingly with respect to the NSRR probe position. The system shows no hysteresis, and each resonance frequency corresponds to a specific displacement level, so a one-to-one mapping between the displacement and the frequency shift is viable. An important feature of the sensor is that the NSRR probe is separated into two movable parts that are electrically shorted by a cable on the upper outermost tooth (shown in Figure 1a), which makes the sensor free of the necessity of strain propagation to the chip, and thus, the sensor is not bounded by the strain limits dictated by the elastic modulus of the NSSR probe material. Since we select the initial separation between the two parts of the NSRR, we can also acquire the strain information by either calculating it from the measured displacement or by using a calibration with strain gauges performed before the installation of the sensor. The maximum monitoring distance, *D_m_*, which is the distance between the NSRR probe and the antenna, is observed at around 80 cm in the experiments. However, as expected, as *D_m_* is increased, the resonances become harder to detect and the distinguishability is reduced.

In this paper, we demonstrate that the entirety of the elastic and plastic deformation regions can be telemetrically measured by the wireless and passive metamaterial-based sensor. Here, as a proof-of-concept demonstration, the NSRR probe is attached to a standard (8-mm diameter) steel reinforcing bar (rebar), and the results are found to be highly accurate when they are compared with the data collected by strain gauges in the elastic region and by an extensometer in the plastic region. To the best of our knowledge, this is the first account in the wireless sensing literature that shows the telemetric recording of the elastic-plastic deformation of a standard steel rebar, which is pulled by a high-scale loading setup to the level of breaking. This is especially important when it is considered that available technologies, like strain gauges, are not able to withstand this level of high strain and break up at much lower displacement levels than those reached in this study. It is also shown that the sensor is still able to work in the presence of the concrete cover, which is known for attenuating the microwave signal. The presence of the steel rebar behind the NSRR probe and the concrete cover between the antenna and the probe makes the proposed measurement environment similar to a real-life scenario. Additionally, we test the sensing system for different types of misalignment scenarios that can occur during the operation of the sensor when the rebar is subject to high levels of force and discuss their effects on the performance. The sensing system exhibits an ability to monitor and evaluate deformation both at low (μm) and high (a few mms) levels of displacement successfully with a high resolution, all the way up to the breaking point of the rebar.

## 2. Experiments

### 2.1. Elastic Region Strain Experiments

Detailed information about the architecture and working principles of the sensor can be found in [22]. In [22], we demonstrated that the sensor is capable of functioning accurately at two different measurement setups: firstly, at the translation stage, where a controlled displacement is created between the two parts of the NSRR, and secondly, at a high-force mechanical setup, where it is attached to a rebar, which is pulled with a force acting along the vertical direction. The elastic-region stress-strain curve obtained by the sensor with a calibration based on the average strain measured by three strain gauges from an initial experiment was shown to be in a good agreement with the gauges for all other measurements.

The first set of the experiments that forms the topic of this paper is performed in a similar high-force setup. This setup is shown in Figure 2a. An 8-mm diameter standard steel rebar is clamped between the two jaw faces in the vertical position, and the NSRR probe parts are fastened on the rebar, as seen in the picture. Then, by applying force, the rebar is elongated and released in its elastic (linear) region, where no permanent deformation is observed and where the strain forming on the rebar is linearly proportional to the stress. The applied force is increased from 0 to 1350 kgF and again decreased to zero linearly in a repetitive fashion for three cycles. Stress is calculated by dividing the applied force to the cross-sectional area of the rebar. The monitoring distance *D_m_* is 10 cm. In the experiments, three strain gauges are utilized for comparison purposes. The strain gauges and the NSRR probe are placed around the rebar at the same elevation, but with a 90° angle from each other. Since the rebar is under constant axial stress, the average of the strain values retrieved from two opposing gauges, which are next to the NSRR probe, is assumed to be comparable with the average strain read from the sensor and the strain value read from the gauge opposing it. Therefore, for the experiment, the strain value corresponding to the frequency shifts for every time instant is found with this method. This experiment provides a way of calibration; and for all other experiments, this transformation between the frequency shift and strain is used to evaluate the sensor data.

The strain information obtained from the sensor and the average of the strains by the strain gauges are plotted *versus* time in Figure 2b. The frequency shift utilized to obtain this graph is found by tracking the minimum points of the shifting dips in the frequency domain after applying a polynomial fit to each peak in order to minimize the effect of system noise. In this experiment, due to the large additional capacitance introduced by the rebar, the NSRR resonance frequency drops from 400 MHz to the 289-MHz level. The minima of the shifting dips are observed to be varying from 288.5 to 289.1 MHz at each cycle. This is a slight change, corresponding to a maximum frequency variation of 0.208% compared to the operation frequency of 289 MHz. This frequency shift information is converted to microstrain by using the calibration method described above. It is observed that both the sensor and strain gauge data agree very well for the whole measurement range, *i.e.*, part of the linear region that extends up to about 1200 microstrains. This experiment demonstrates that the sensor shows no hysteresis-type behavior, and a specific frequency peak always corresponds to the same strain level, regardless of the applied force regime. This is important, because this way, we can make a one-to-one mapping from the resonance frequency to the strain or displacement or *vice versa*.

The resolution of the sensor in translation stage measurements was demonstrated as better than 1 μm in [22]. For the elastic region experiment, the strain measured by the sensor is theoretically the additional displacement due to applied force divided by the original length between the attachment points of the NSRR probe, which is the midpoints of the two sensor parts and is equal to 2.35 cm. Therefore, the displacement resolution of 1 μm achieved in translation stage measurements corresponds to a strain resolution of 1 μm/2.35 cm, that is 42.5 microstrain. When Figure 2b is investigated closely, the minimum sensor measurement steps are observed to vary between 100 and 200 microstrains. This shows that the sensor resolution degrades as a result of the noise introduced by the environmental effects and the effect of the rebar placed behind the NSRR probe in the setup.

### 2.2. The Effect of Concrete

**Figure 3.**
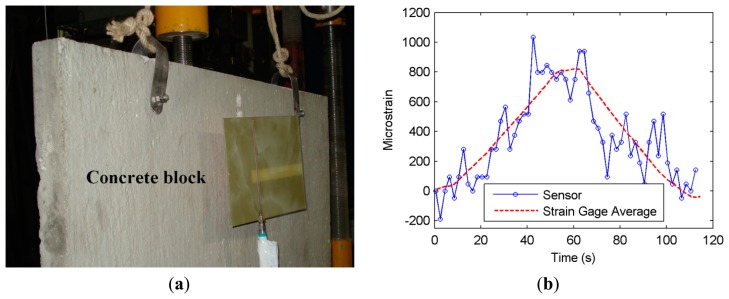
(**a**) Elastic (linear) region pulling and releasing experiments repeated with a concrete block of 4 cm thickness; (**b**) strain read by the metamaterial-based sensor and average strain gauge data.

In a real-life scenario, it is frequently the case that a concrete cover is present between the antenna and the sensing structure located on the rebar, which is the NSRR probe in this study. It has previously been shown that concrete attenuates the RF signals due to its loss factor [25,26]. To see the sensor performance in this case, a concrete block of 4 cm in thickness, which represents the typical clear cover of a reinforced concrete member, is placed between the antenna and the rebar on which the NSRR probe is attached, and the linear region strain measurement is repeated. The force applied by the setup is increased linearly from 0 to 900 kgF and again decreased to a zero in one cycle. The measurement setup and the microstrain obtained by the sensor and the strain gauges when the concrete block is placed between the antenna and the probe are shown in Figure 3, respectively. It is observed in Figure 3b that the sensor can still track the strain in the presence of the concrete throughout the whole range, despite the degradation of sensor resolution due to the increased level of noise. The data of Figure 3b is the raw data from the measurements, where no post-processing is applied to exhibit the worst case scenario due to noise. Fluctuating components can be eliminated by several methods, e.g., simply by averaging or employing a low-pass filter. The calibration method used in the transformation of the frequencies to strain is the same as the case without the concrete. A successful transformation using the same calibration for the no-concrete case also proves that a typical concrete cover does not constitute a significant attenuation on the near-field interaction between the antenna and the NSRR probe. This is a promising result, especially when it is considered that this scenario includes the effects of both the rebar and the concrete block on the sensing system. In a real-life scenario, the effect of the concrete cover and the reinforced concrete behind the NSRR on the sensor will be in the form of decreasing the resonance frequency of the coupled system even further by the large capacitance brought in by this medium, which is an amplified version of the case of introducing a single rebar. This effect can be dealt with by employing different calibrations for each medium. This way, a different strain-frequency shift mapping is obtained for each case.

### 2.3. Plastic Region Displacement Experiments

**Figure 4.**
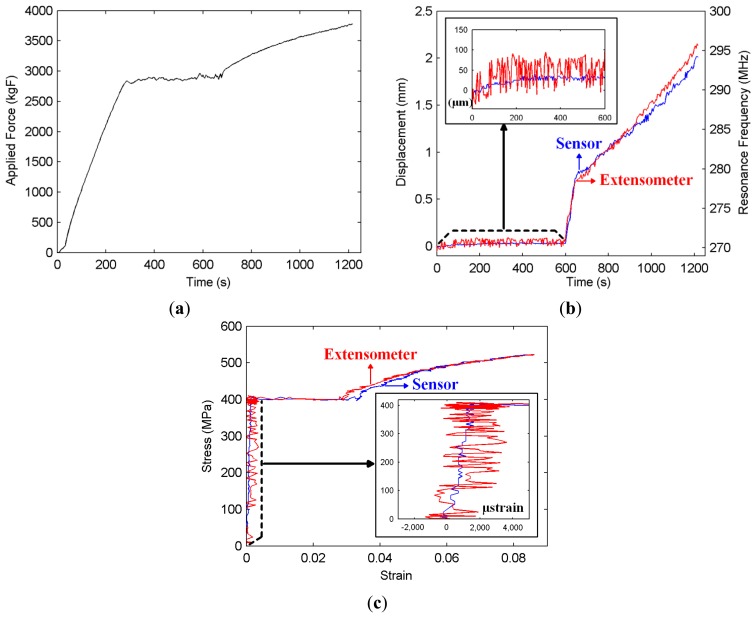
Plastic region strain and displacement measurements: (**a**) applied force (in kgF); (**b**) displacement information acquired from the metamaterial-based sensor and from the extensometer. Corresponding resonance frequencies of the sensor are also given on the right vertical axis. Top left: Zoomed elastic region (inset); (**c**) Stress (in MPa) *versus* the calculated strain acquired from the sensor and from the extensometer. Bottom right: Zoomed elastic region (inset).

The measurement of the plastic region displacement and strain is also important in terms of understanding the level of damage during an overloading or an earthquake, which may lead to a higher level of displacements and strains. It has been shown in [22] that the sensor can track displacements as large as 20 mm, and this value is more than enough to be able to detect the displacement and strain levels that can be experienced in a reinforced concrete member prior to strength degradation. In such a plastic deformation experiment, the level of the applied force is increased to the degree that the strain forming on the steel rebar exceeds the elastic region limit. The force continues to be increased past the yield stress, which signals the initiation of irreversible plastic deformation. Around this point, yielding is observed, where the stress level stays more or less the same, while the strain is increasing. Following this, strain hardening is initiated, where the stress-strain relationship becomes nonlinear and the ultimate tensile stress, which is the maximum stress level that can occur on the rebar, is measured. If the force continues to be applied to the rebar, necking occurs, and the rebar is fractured and torn into two pieces. Important stress points, such as yield and ultimate tensile stress and the corresponding strains are known for standard steel rebars used in structures. Therefore, the ability to measure strain provides valuable information about the possible physical damage in a structural member. The following experiment is performed in this plastic region where the force is increased to 3750 kgF (see Figure 4a), which leads the steel rebar to reach to its tensile strength point at about 500 MPa (see Figure 4c). Although strain gauges function quite efficiently and without any significant noise in the elastic region, they cannot be used in the plastic region measurements, typically starting to break and to come off after a certain strain level (about a few thousand microstrains). For this reason, for the plastic region measurements, extensometers are commonly used for comparison purposes. The extensometer used in these experiments is shown in Figure 2a.

The applied force reading obtained from the data acquisition system is presented as a function of time in Figure 4a, while the displacement readings from the sensor and the extensometer are plotted in Figure 4b. The displacement values used in this curve are the additional displacements to the distance fixed between the midpoints (attachment points to the rebar) of the two parts of the NSRR, which is 2.35 cm. Finally, the stress-strain curves obtained from the sensor and extensometer data are presented in Figure 4c. The elastic region (t = 0–600 s in Figure 4a), yielding region (t = 600–660 s) and the strain hardening region (t > 660 s) can all be seen in Figure 4b. A fast increase in the displacement from a small elastic displacement value to around 0.9 mm is observed during yielding (see Figure 4b). In the yielding region, the rebar cannot carry the applied load, and thus, this fast deformation continues until it restores its load carrying capacity due to strain hardening. After that point (660th second), the displacement starts to increase in a linear fashion to 2 mm. Figure 4b and c reveals that the agreement between the sensor and extensometer data is quite high. The wireless sensor readings follow the displacement data of the extensometer very closely. Here, it should also be noted that in Figure 4b and c, the noise present on the wireless sensor data is observed to be much weaker compared to that of the extensometer in the elastic region; and in the plastic region, their noise levels are more or less the same. In Figure 4b, at the right vertical axis, the measured resonance frequencies of the sensor are also shown.

As previously mentioned, there is one-to-one mapping between the displacement and the corresponding resonance frequency. To relate the resonance frequency shift to the displacement, we use a numerical fit derived from the extensometer displacement values read in the experiment that serves as a calibration. The numerical fit is chosen as an exponential-type of function, so that it reflects the nonlinear characteristics of the sensor that come into play for large displacements. Strain is calculated by dividing the sensor displacement readings by the original length of 2.35 cm, which is the initial distance between the two attachment points of the NSRR parts. The strain corresponding to the extensometer readings is also calculated similarly, where the displacement values are divided by the original separation between the extensometer arms, which is 4.7 cm in our case. It should be pointed out that this value is twice the original sensor attachment point distance of 2.35 cm, since the extensometer is placed such that its arms touch the sensor from both sides, as shown in Figure 2a. Stress is calculated by dividing the applied force to the cross-sectional area of the rebar. The effect of the concrete cover in terms of the increased system noise is less evident in the plastic region measurements, since the shifting range is much higher and the peaks can more easily be distinguished; therefore, the sensor is less prone to noise in that region. For this reason, as a representative case, only the elastic region measurements with the concrete cover are shown.

## 3. Resilience to Misalignments

**Figure 5.**
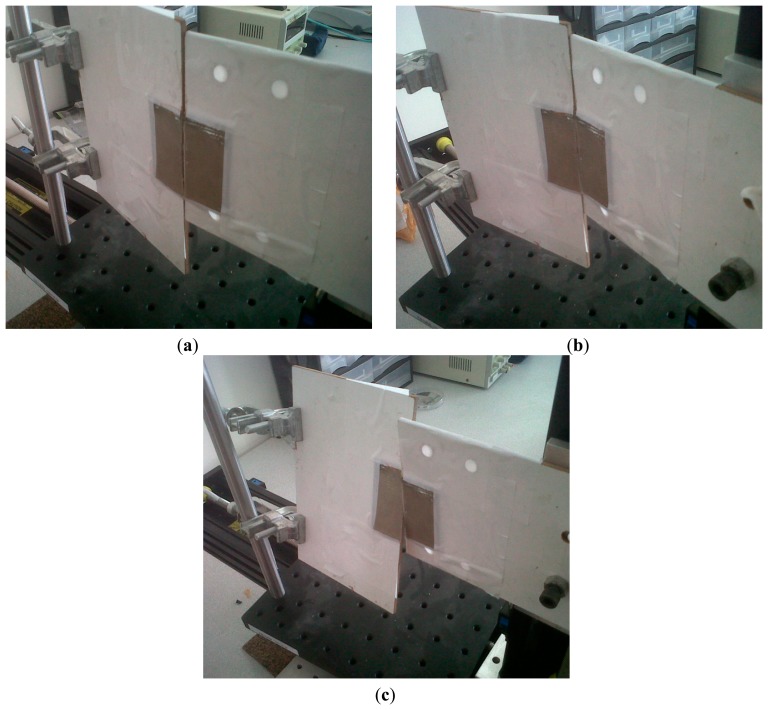
Possible misalignments scenarios of the sensor: (**a**) inward bending of the NSRR probe parts; (**b**) outward bending of the NSRR probe parts; (**c**) twisting of the NSRR probe parts.

**Figure 6.**
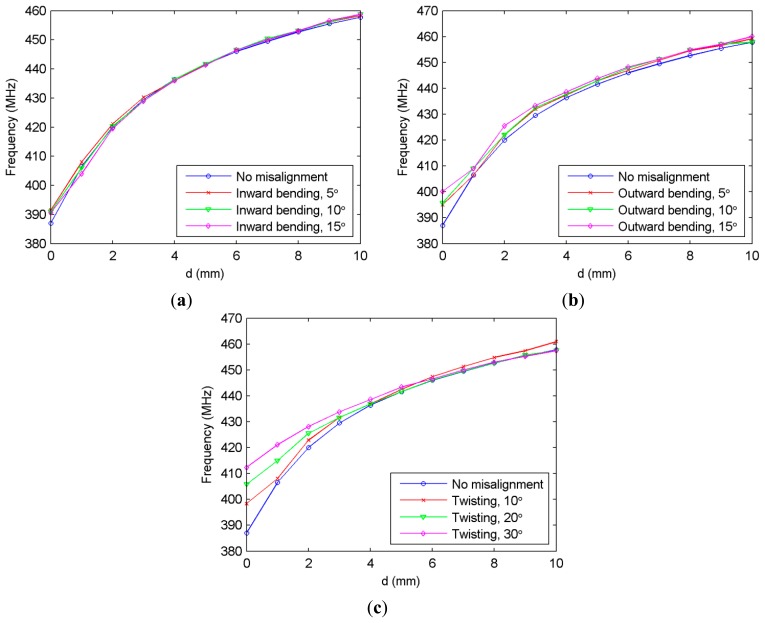
Shifting frequency of the metamaterial-based sensor *versus* displacement using the data experimentally obtained for possible misalignments: (**a**) inward bending of the NSRR parts, given for the angles of 5°, 10° and 15°; (**b**) outward bending of the NSRR parts, given for the angles of 5°, 10° and 15°; (**c**) twisting of the NSRR parts, given for the angles of 10°, 20° and 30°.

The robustness of the wireless metamaterial-based sensor is also tested on a translation stage to identify to what extent possible misalignments affect the readings. Among these misalignments are bending of the front faces of the two NSRR parts (faces with metal strips) towards each other (inward bending, see Figure 5a) and away from each other (outward bending, see Figure 5b). Yet another possible type of misalignment is twisting of the rebar, which causes the two NSRR parts to rotate in opposite directions around the rebar (see Figure 5c). In Figure 6a, the frequency shift of the sensor on the translation stage *versus* a created NSRR displacement (d) is presented for the cases when there is no misalignment and when there is an inward bending with the angles of 5°, 10° and 15°. The same characterization is shown for several angles in the case of outward bending in Figure 6b and in the case of twisting in Figure 6c.

The first two figures show that the bending of the NSRR parts does not have a major effect on the sensor operation, since the curves obtained for these misalignments are no different than that without misalignment. The only possible discrepancy is experienced when d = 0 mm for the outward bending, since the resonance frequency difference between the case of no misalignment and the case of 15° bending is observed to be over 10 MHz. For twisting, the resulting deviation from the case of no misalignment is higher for smaller displacements, but as d increases, this deviation is reduced. It should also be emphasized that the angles of 10°, 20° and 30° are actually fairly extreme angles for such a twisting in practice, and they were selected to test the worst possible cases. Nevertheless, especially for d = 0 mm, the resonance frequency difference between the cases of no misalignment and twisting is quite high. This problem can be addressed by allowing an initial separation (d) between the two NSRR parts, e.g., 1 mm, and choosing this as the starting displacement. Although this comes at the cost of reduced sensitivity and dynamic range of the sensor (which is still quite high), it increases the robustness.

## 4. Conclusions

In this work, a metamaterial-based strain and displacement sensing system is shown to accurately record data in both elastic and plastic deformation regions for a standard 8-mm diameter steel construction rebar, which is elongated along its vertical axis. The sensor data is compared with the data collected from the strain gauges in the elastic region and with the data collected from an extensometer in the plastic region. The results are found to agree well in both regions. The measurements are repeated for the case where a 4 cm-thick concrete block is placed between the antenna and the NSRR, showing that the sensor can still function properly. Several possible misalignment scenarios are investigated, and their effects on the sensor performance are discussed. These experiments performed on the proposed sensor reveal that this approach is promising for detecting strain and displacement in SHM, as well as the damage assessment of reinforced concrete members.
